# Failed treatment of large adenomyosis with penetrating glandular distribution using hysteroscopic LNG-IUD suture fixation: a case report and literature review

**DOI:** 10.3389/fmed.2026.1762481

**Published:** 2026-03-25

**Authors:** Wenhui Wang, Wenhua Liu, Gensheng Wang, Liang Qian

**Affiliations:** 1 Department of Pathology, Hangzhou Women’s Hospital (Hangzhou Maternity and Child Health Care Hospital), Hangzhou, Zhejiang, China; 2 Department of Obstetrics and Gynecology, Anqing Hospital Affiliated to Anhui Medical University, Anqing, Anhui, China; 3 Department of Gynecology, Hangzhou Women’s Hospital (Hangzhou Maternity and Child Health Care Hospital), Hangzhou, Zhejiang, China

**Keywords:** adenomyosis, hysteroscopic, laparoscopy, LNG-IUD, magnetic resonance imaging

## Abstract

**Introduction:**

Hysteroscopic LNG-IUD suture fixation is an innovative procedure for treating adenomyosis, effectively addressing the issue of expulsion. However, this case report presents a failed outcome and offers an exploratory summary of the indications for LNG-IUD.

**Materials and methods:**

This case report describes a 38-year-old woman experienced a relapse of adenomyosis 1 year after undergoing a hysteroscopic Mirena suturing procedure. A comparison of MRI images taken before and 1 year after the placement of the Mirena revealed a diffuse distribution of endometrial tissue on the posterior uterine wall. Despite the Mirena’s placement, the uterus continued to enlarge progressively. The adenomyosis lesion was excised laparoscopically, and the Mirena device was reinserted.

**Results:**

The postoperative course was uneventful. At a follow-up 1 year later, the patient reported no dysmenorrhea and only occasional, intermittent vaginal bleeding. This case report highlights the limitation of LNG-IUS in treating advanced adenomyosis, even with suture fixation.

**Conclusion:**

Hysteroscopic LNG-IUD suture fixation is not recommended as a long-term treatment option for patients with extensive adenomyosis characterized by glandular penetrating distribution. Although this procedure can prevent device detachment and temporarily alleviate symptoms, it does not offer a durable solution for such severe cases.

## Background

Adenomyosis is characterized either as a diffuse or localized lesion that results from the presence of endometrial glands and stroma within the myometrium. This condition predominantly affects women of childbearing and perimenopausal ages, leading to symptoms such as heavy menstrual bleeding, painful menstruation, and reduced reproductive function, all of which significantly impair quality of life (1). The levonorgestrel-releasing intrauterine system (LNG-IUS) was initially used as a contraceptive device, but the localized high levonorgestrel environment in the uterine cavity can effectively reduce menstrual bleeding and relieve dysmenorrhea through direct action on the endometrium. As a result, the LNG-IUS is now extensively utilized in the treatment of adenomyosis, with substantial therapeutic outcomes reported in multiple studies (2–4).

Nonetheless, the LNG-IUS is not suitable for all patients, and its use is constrained by the absence of standardized guidelines (5). One significant factor compromising treatment efficacy is the potential expulsion of the LNG-IUS, which can be precipitated by the enlarged uterus and the flushing effect of heavy menstrual flow (6, 7). To counter this issue, the hysteroscopic cold knife system employs sutures to secure the device within the uterine cavity, thereby preventing expulsion (8, 9). However, is securing the LNG-IUS to prevent expulsion always sufficient to achieve desired outcomes? We present a case where the treatment was unsuccessful even after the Mirena suture procedure. Additionally, we conduct a literature review and discuss the indications for using the LNG-IUS.

## Case presentation

A 38-year-old nulliparous woman was admitted to the hospital on December 1, 2022, due to intermittent vaginal bleeding persisting for 2 months and lower abdominal pain lasting 1 week. The patient was 170 cm in height and weighed 96 kg, with a body mass index (BMI) of 33.2 kg/m^2^. Fifteen years before her admission, she had undergone laparoscopic surgery to excise bilateral ovarian endometriomas, followed by 6 months of therapy with GnRH-a (gonadotropin-releasing hormone agonist). Despite planning for pregnancy, the patient was unable to conceive successfully. She experienced an ectopic pregnancy, resulting in the surgical removal of the affected fallopian tube.

Five years ago, the patient was diagnosed with adenomyosis, presenting with dysmenorrhea and increased menstrual flow. She underwent high-intensity focused ultrasound (HIFU) treatment to alleviate her symptoms. However, 2 years later, due to persistent severe dysmenorrhea and heavy menstrual bleeding, she required a laparotomy to excise adenomyosis lesions. Unfortunately, the adenomyosis recurred 2 years after the surgery, leading her to undergo a hysteroscopic Mirena suturing procedure at our center.

**Figure 1:**
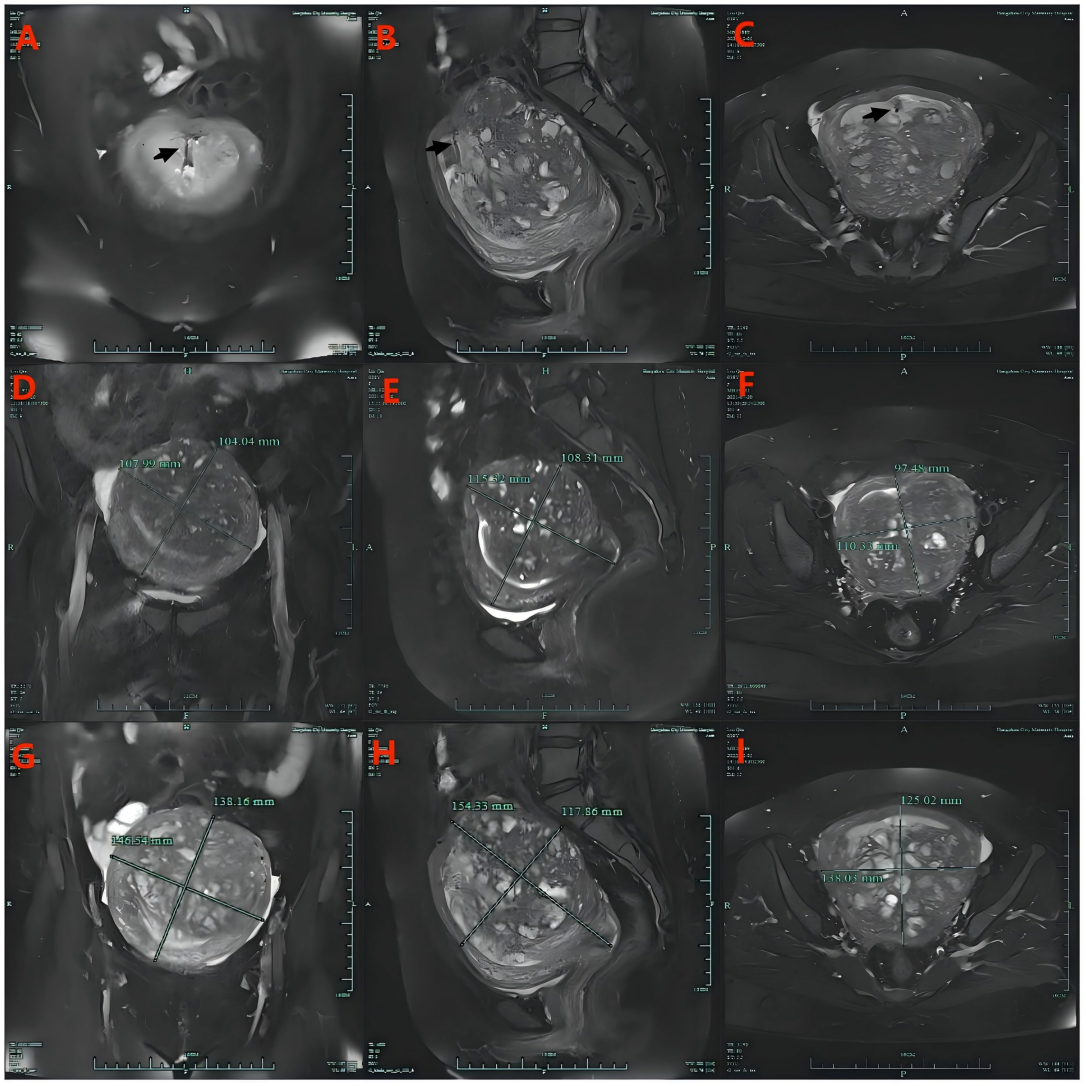
The Mirena is visible in **A–C** (black arrow). **(D–F)** Are magnetic resonance images of the uterus in coronal, sagittal, and transverse planes, respectively, before Mirena fixation. **(G–I)** Are the magnetic resonance images of the uterus in coronal, sagittal, and transverse planes, respectively, 1 year after Mirena fixation.

Postoperatively, the patient experienced amenorrhea for 1 year and showed improvement in her dysmenorrhea symptoms. However, 2 months prior to her admission, vaginal bleeding recurred. A comparison of MRI images taken before and 1 year after the placement of the Mirena revealed a diffuse distribution of endometrial tissue on the posterior uterine wall. Despite the Mirena’s placement, the uterus continued to enlarge progressively, as shown in Figure 1. Before fixation, the maximal uterine dimensions were approximately 115.32 × 108.30 × 110.33 mm (longitudinal × anteroposterior × transverse), corresponding to an estimated uterine volume of about 720.7 cm^3^ using the prolate ellipsoid formula (L × AP × T × 0.523) (10). One year after fixation, the uterus further enlarged to approximately 154.33 × 138.16 × 146.54 mm, with an estimated volume of about 1634.1 cm^3^, representing an increase of approximately 127%.

**Figure 2:**
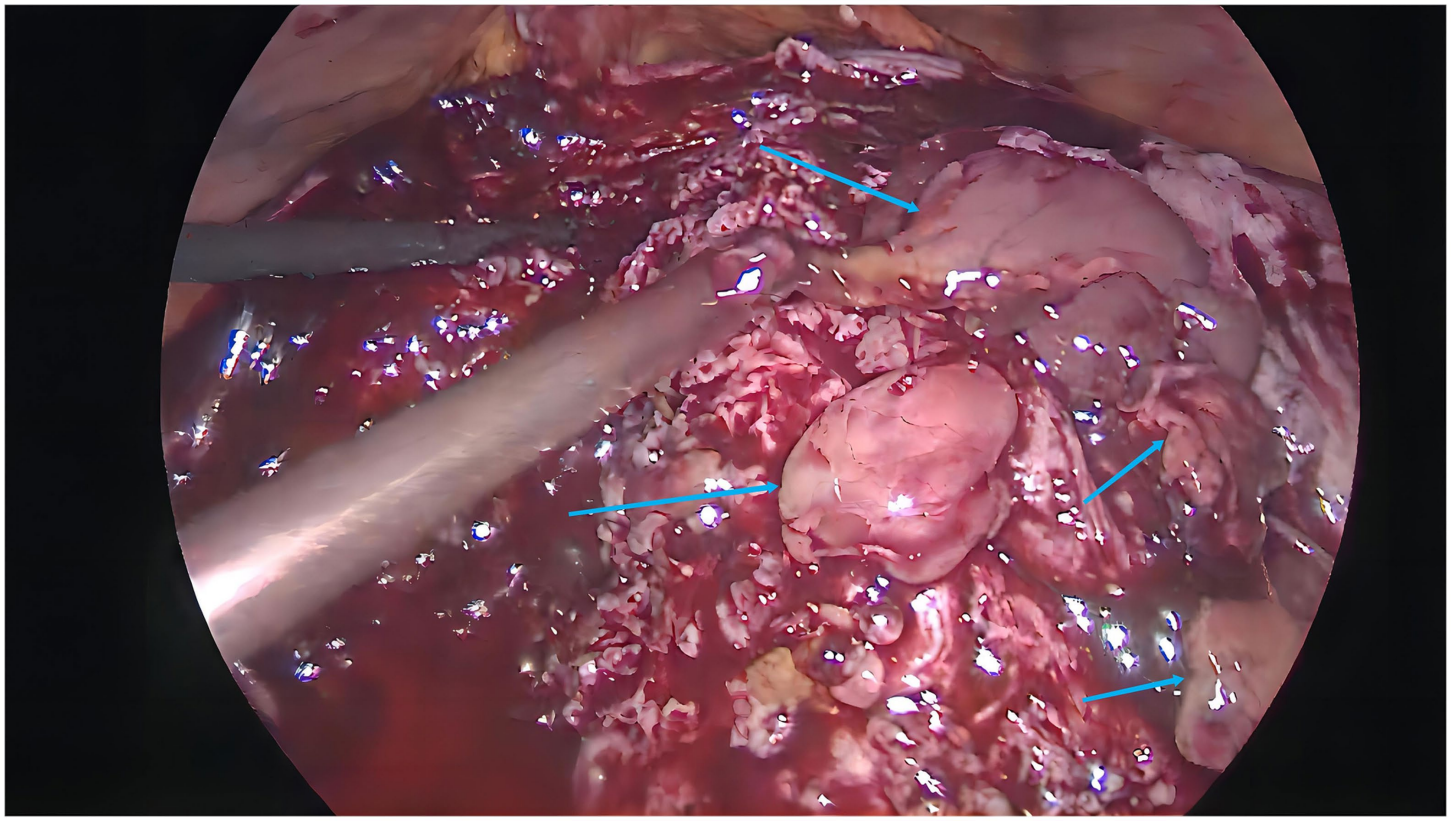
Ectopic endometrial glandular hyperplastic tissue is seen in the myometrium (blue arrow).

**Figure 3:**
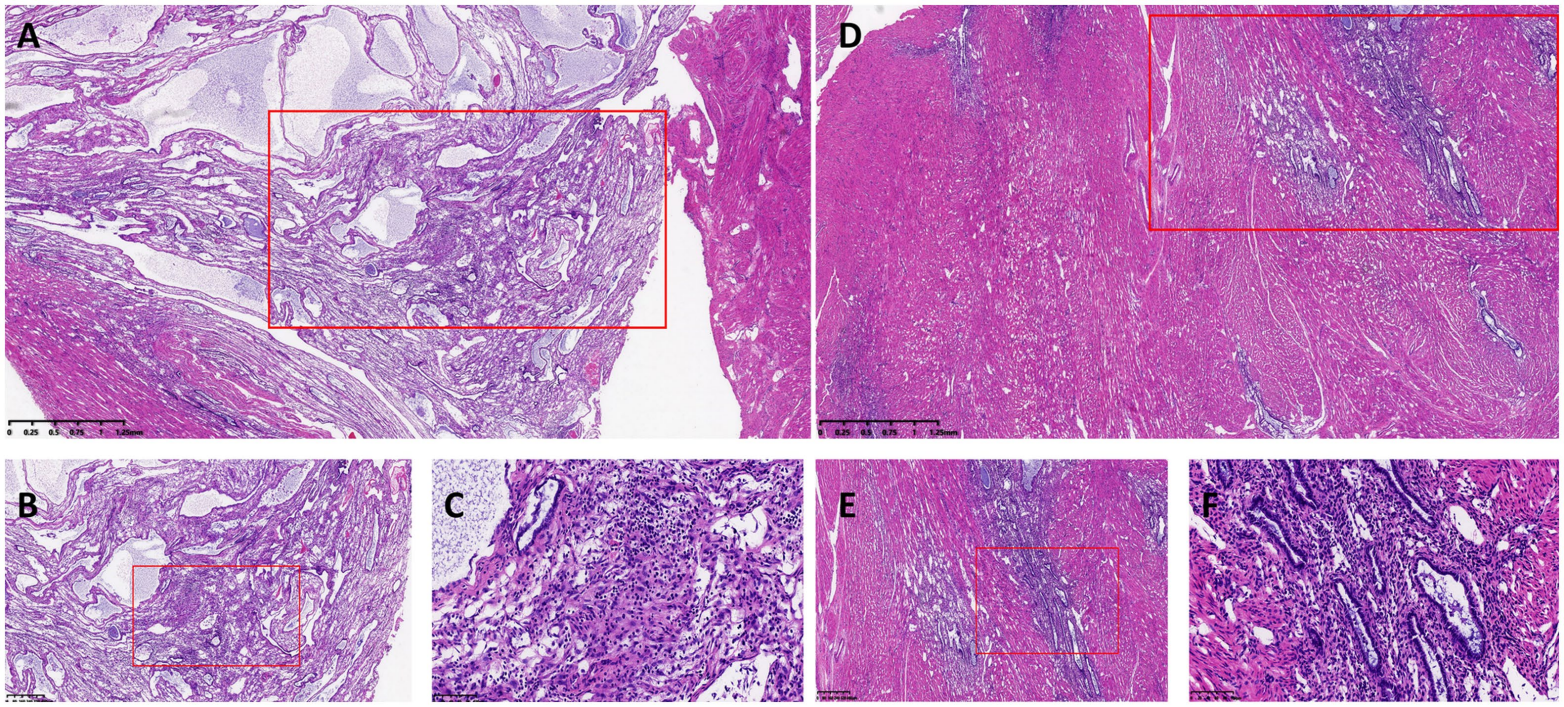
Histopathological comparison between inner and outer myometrial adenomyotic lesions (hematoxylin and eosin staining). **(A–C)** Adenomyotic lesions located in the inner myometrium adjacent to the endometrium. **(A,B)** Low- and intermediate-power views demonstrate ectopic endometrial glands within smooth muscle bundles. The boxed area indicates the region selected for higher magnification. **(C)** Higher magnification of the boxed area in **B** shows glandular atrophy, flattened epithelium, decidual-like stromal change, and focal chronic inflammatory infiltration. **(D–F)** Adenomyotic lesions located in the outer myometrium adjacent to the serosa. **(D,E)** Low- and intermediate-power views reveal proliferative-phase-like ectopic endometrial glands. The boxed area indicates the region selected for higher magnification. **(F)** Higher magnification of the boxed area in **E** demonstrates uniformly shaped glands lined by single-layer cuboidal epithelium and densely cellular stroma.

Upon admission, the patient was initially treated with cefoxitin for infection control over a period of 1 week. At the time of treatment decision-making, the patient expressed a desire for future fertility. However, she was counseled that, given the extent of adenomyosis and her clinical history, the likelihood of successful pregnancy was considered to be low. Despite this, the patient strongly wished to preserve her uterus. In addition, she declined long-term oral medical therapy, given her obesity status and concerns regarding prolonged systemic hormonal treatment. Laparoscopic excision of adenomyosis lesions was subsequently performed. During the surgery, extensive glandular hyperplasia was observed within the muscular layer, as depicted in Figure 2. Postoperatively, the Mirena was reinserted into the uterine cavity. The patient’s recovery was smooth. Histopathological examination of the surgical specimen confirmed adenomyosis. Notably, distinct morphological differences were observed between lesions located in the inner and outer myometrium. Adenomyotic foci adjacent to the endometrium exhibited glandular atrophy, stromal edema, and decidual-like changes, whereas lesions in the outer myometrium demonstrated proliferative-phase–like endometrial glands with preserved glandular architecture and densely cellular stroma (Figure 3). At a two-year postoperative follow-up in 2024, the patient reported no dysmenorrhea or abnormal uterine bleeding. However, during a subsequent follow-up in 2025, she reported recurrence of adenomyosis-related symptoms and had undergone hysterectomy at an external hospital. A detailed timeline of the patient’s clinical course is summarized in Table 1. Written informed consent for publication was obtained from the patient.

**Table 1:** Clinical timeline.

Year	Key clinical findings/Diagnosis	Intervention	Outcome/Follow up
2007	Bilateral ovarian endometriomas	Laparoscopic cystectomy; GnRH-a therapy for ~6 months	Symptom relief; later infertility reported
2009	Ectopic pregnancy	Salpingectomy of one side	Failure to conceive
2017	Adenomyosis diagnosed; dysmenorrhea and heavy menstrual bleeding	High-intensity focused ultrasound (HIFU)	Temporary symptom improvement
2019	Persistent/recurrent severe dysmenorrhea and heavy menstrual bleeding	Laparotomy for adenomyosis lesion excision	Symptom improvement
2021	Recurrent adenomyosis with enlarged uterus; high risk of LNG-IUS expulsion	Hysteroscopic LNG-IUS (Mirena) suture fixation	Amenorrhea and symptom relief for 1 year
2022	Recurrent adenomyosis	Laparoscopic excision of adenomyosis lesion; LNG-IUS reinserted	Postoperative course uneventful
2024	Follow-up after laparoscopic excision	Outpatient follow-up	Mirena in place, no dysmenorrhea; occasional intermittent vaginal bleeding
2025	Patient-reported recurrence of adenomyosis	Hysterectomy at an external hospital	Definitive treatment

## Discussion and conclusion

The levonorgestrel-releasing intrauterine system (LNG-IUS) is widely used in gynecological practice. It has demonstrated favorable safety profiles and promising oncological outcomes in selected women with early-stage endometrial atypical hyperplasia or endometrial cancer who are unfit for surgery (11). In addition, LNG-IUS has been increasingly applied in the management of adenomyosis due to its local progestogenic effects. Although studies evaluating the effectiveness of the LNG-IUS in treating adenomyosis generally affirm its benefits, these studies often lack proper screening and categorization of patients with adenomyosis. Additionally, there is a scarcity of research on individuals who experience poor outcomes with the LNG-IUS, leaving the factors that determine its effectiveness somewhat ambiguous. Hysteroscopic suture-fixation of the LNG-IUS is particularly suited for patients with a history of expulsion or those at high risk of displacement, many of whom are in the later stages of adenomyosis. However, the therapeutic efficacy of the LNG-IUS in such cases remains uncertain due to the potential expulsion of the device. Suture fixation secures the LNG-IUS within the uterine cavity, mitigating the issue of expulsion and allowing for a more accurate assessment of its efficacy in patients with advanced adenomyosis.

The MRI of the patient in this case revealed a penetrating subtype of adenomyosis, according to the classification system proposed by Han et al. (12). The adenomyotic lesions were confined to and had fully penetrated the entire posterior wall of the uterus. Additionally, diffuse ectopic glandular tissue with cystic dilatation was evident in the myometrium of the posterior uterine wall. Within 1 year following the suture fixation, there was a marked improvement in the symptoms of uterine bleeding and dysmenorrhea. However, the uterus remained noticeably enlarged, and the glandular structures in the myometrium were more prominently dilated.

The precise mechanism by which the LNG-IUS functions in adenomyosis remains unclear. Research indicates that the LNG released locally results in endometrial concentrations that are 1,000 times higher than those found in the bloodstream (13). This high local concentration of LNG is believed to induce decidualization and atrophy of the endometrium, reduce endometrial blood flow, and suppress estrogen receptor (ER) expression (14, 15). Additionally, it has been documented that the LNG-IUS can reduce the size of adenomyosis lesions (4).

We speculate that the improvement in the patient’s symptoms within 1 year postoperatively can be attributed to the high concentrations of LNG acting on the endometrium and the junctional zone within the uterine cavity. However, due to significant thickening of the posterior uterine wall, the high concentration of LNG was unable to penetrate the deep myometrium’s microenvironment. Consequently, the ectopic glandular tissue within the myometrium remained unaffected and continued to expand, leading to an ongoing enlargement of the uterus. The area of the endometrium also increases as the uterus enlarges. Furthermore, the concentration of LNG in the uterine cavity may decrease over time. Once the LNG concentration becomes insufficient to counteract the enlargement of the uterus and the expanding endometrial surface, the patient’s symptoms are likely to recur. Notably, histopathological examination in this case revealed spatial heterogeneity between adenomyotic lesions located near the endometrial surface and those in the outer myometrium. Lesions adjacent to the endometrium exhibited glandular atrophy and decidual-like stromal changes consistent with progesterone exposure, whereas deeply located lesions demonstrated proliferative-phase-like morphology without evident progesterone-related suppression. This differential response may reflect limited penetration of locally released LNG into deeply infiltrating adenomyotic tissue and provides pathological support for the proposed mechanism. In a study of uterine histopathology in women who underwent hysterectomy after failed of Mirena treatment, it was found that the stroma and glands of deep adenomyosis did not show a progesterone response, some adenomyotic foci exhibited a proliferative patter (16). Another study indicated that the maintenance of LNG in the myometrium results from absorption into the systemic circulation from the uterine cavity, rather than direct diffusion, as LNG levels in the myometrium of patients treated with Mirena did not differ from those in patients after taking 250 μg of oral LNG (17). These findings support our hypothesis. However, it should be emphasized that these findings are largely based on theoretical speculation and observations from a single case. As an inherent limitation of a case report, the conclusions drawn from this study have limited generalizability and should be interpreted with caution. Further clinical studies with larger sample sizes, as well as basic experimental investigations, are warranted to validate the proposed mechanisms and to better define the role of hysteroscopic LNG-IUS suture fixation in the management of advanced adenomyosis.

In conclusion, while hysteroscopic LNG-IUS suture fixation can prevent device detachment and may improve symptoms in the short term, it may not represent an optimal long-term treatment option for patients with extensive adenomyosis characterized by glandular penetrating distribution. These observations suggest that caution is warranted when considering this approach in advanced disease, and individualized treatment strategies should be carefully evaluated.

## Data Availability

The original contributions presented in the study are included in the article/supplementary material, further inquiries can be directed to the corresponding author.
